# Granular cell tumour of the soft tissues: a case report and literature review

**DOI:** 10.1186/1477-7800-3-21

**Published:** 2006-08-24

**Authors:** NA Qureshi, M Tahir, AR Carmichael

**Affiliations:** 1 Department of General and Breast Surgery, Russells Hall Hospital, Dudley, West Midlands, UK

## Abstract

Granular cell tumours (GCT) of the soft tissues are rare benign tumours but some time may be difficult to distinguish from malignant neoplasms. It is important that clinicians are aware of their existence. We present a new case of GCT of the soft tissues followed by a brief review of literature.

## Background

Granular cell tumours are rare benign tumours and were first described by Weber in 1854 [1]. Abrikossoff, in his publication of 1926 [2] reported the occurrence of granular cell tumour in the tongue. He initially proposed that these tumours originate from striated muscle cells and as such referred to them as "myoblastomas." Subsequent evidence has indicated that these tumours are most likely derived from Schwann cells of the peripheral nerves [3,4]. We describe a new case of soft tissue granular cell tumour in a 47-year-old woman, with a brief review of the literature.

## Case report

A 47-year-old woman presented to breast clinic with a swelling over sternal region that was causing discomfort. On physical examination, there was a firm, mildly tender, 5 × 4 cm palpable lump in the midline, anterior to sternum. Overlying skin was normal and mobile. Fine needle aspiration of the lesion revealed only blood. Ultrasound guided core biopsy of the lesion showed sheets of cells with abundant granular cytoplasm and small unremarkable bland nuclei. A diagnosis of granular cell tumour was made. No evidence of malignancy was noted on the core biopsy. The patient underwent a wide surgical excision of the tumour and had an unremarkable post-operative recovery. She was discharged the next day.

**Figure 1:**
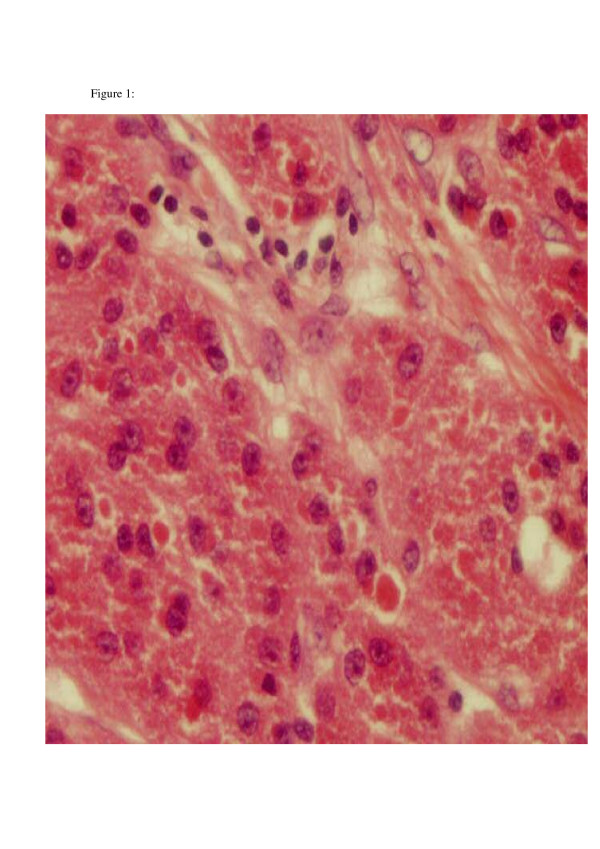
High power of large cells with granular cytoplasm.

**Figure 2:**
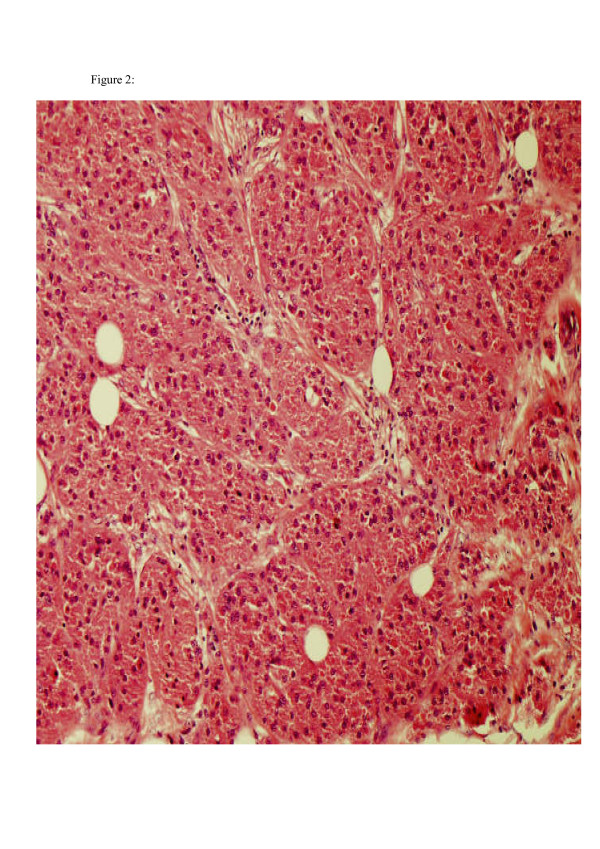
Low power of large cells with granular cytoplasm.

The specimen submitted to pathology contained an irregular piece of soft tissue measuring 5 × 4.5 × 3 cm. The specimen weighed 27 grams. On sectioning, there was a firm nodule, which measured 2.5 cm in diameter and was whitish in colour. The nodule appeared very close to the posterior wall of the specimen. Immunohistoichemistry showed S100 to be diffusely positive, while NSE faintly positive. All these features were suggestive of granular cell tumour. Histology confirmed that excision margins were clear of the tumour. (Histopathology images are shown in figures 1 and 2).

## Discussion

Granular cell tumours are usually asymptomatic and present as smooth, slow-growing, solitary nodules in subcutaneous, intradermal, or submucosal regions. These tumours can arise at any part of the body, but head and neck, chest wall and arms are the three most commonly affected sites. They develop in the breast in about 5% of cases [5]. The overlying skin may be normal, hyperpigmented, or covered with a tuft of hair in the subcutaneous variety. These tumours primarily affect adults but may well be found in children [6]. They are more common in females and often develop between the second and sixth decades of life. These tumours are usually solitary but multiple satellite nodules are present in 10 to 15% of all cases. Although granular cell tumours tend to be benign, there are a few reports of them behaving in a malignant fashion. When granular cell tumours occur in the breast, the clinical and pathologic appearance can closely mimic breast carcinoma [5]. They may co-exist with malignant lesions. Their diagnosis is based mainly on immunohistchemical confirmation.

The origin of granular cell tumours is uncertain. Initially it was thought that they arise from skeletal muscle because of their cytologic resemblance to myocytes [2]. Others argued that there might be a fibroblastic, histiocystic, or undifferentiated mesenchymal cell origin [7]. More recently, based on the evidence that monoclonal antibody KP-1, which recognizes the lysosome-associated glycoprotein CD68, reacts positively with schwannomas and granular cell tumours, it is believed that these tumours arise from Schwann's cells [4]. Also, granular cell tumours cytoplasmically stain for S-100 protein [3], are closely associated with nerves and are often present in distal nerve trunks. All these features support a Schwann's cell origin. Histologically, these tumors are non-encapsulated and the cells are typically grouped in a nest-like pattern, surrounded by thin strands of fibrous tissue. The cells have centrally placed nuclei surrounded by ample, coarsely granular, eosinophilic cytoplasm. Rapid growth and a large size combined with histologic findings of mitotic figures, cellular and nuclear pleomorphism, necrosis, wide cellular sheets, and a spindle-cell structure increases the likelihood of malignancy.

The treatment of choice is radical resection with clear surgical margins. Radiation and chemotherapy are not advised because of the tumour's high degree of resistance [8]. Complete surgical resection is considered curative. Even when negative margins are not obtained, the prognosis is still favourable. Patients with malignant granular cell tumour or granular cell tumour of malignant potential are best managed with wide local excision and regional lymph node dissection [9]. It is advisable to follow up these patients annually to rule out late recurrences.

## Conclusion

Although granular cell tumours are usually benign and slow growing, it is difficult to distinguish them from malignant lesions. Therefore, it is very important that clinicians and pathologists are aware of their clinical and histopathological features.

## Competing interests

The author(s) declare that they have no competing interest.
